# Surgery

**Published:** 1877-01

**Authors:** 


### III. Surgery.

1. *Percussion of the Bones.* Luecke. (*Centralbl. f. Chir.*, 1876, No. 43.)

The percussion of the bones may be employed for a two-fold purpose, viz.: either to ascertain a painful place in the bone, or to diagnose pathological changes in its texture by the alteration of the normal sound of the bone. For the first mentioned purpose the percussion has, long since, been practiced; but we must give it greater attention, since we begin to surgically treat central affections of the bones, before they can injure the neighboring structures. In order to find most accurately the painful spot, we can, for this kind of percussion, use our fingers or small percussion hammers; but we must never omit to compare the result with the sound side, because of the great variation of sensitiveness among patients.

The second object of the percussion of bones is to ascertain an acoustic difference between a diseased and healthy bone. The long bones of the extremities afford the best conditions for this study. The like bones always show the same sound; but the epiphyses give a higher sound than the diaphyses. A recently united fracture indicates by a lower pitch of its percussion sound the occlusion of its central cavity and the presence of an abnormally thick mass of bone. Chronic osteitis of the epiphysis makes its sound lower, while a tibia which had become very porous yielded a remarkably higher sound than the normal tibia of the other leg.

The percussion of the extremities is executed while they are raised from the bed or table.

2. *Cheap Antiseptic Dressing.* Balfour. (*Ed. Med. Journal*, Aug., 1876.)

Dr. B. uses sulphurous acid, one part to twelve of water, as a constant lotion and dressing for wounds. He claims from it alleviation of pain, diminished suppuration, prompt healing, ease of application, and cheapness. Three cases are quoted in endorsement. J. S. K.

